# Advancements and Challenges in Sucralose Determination: A Comparative Review of Chromatographic, Electrochemical, and Spectrophotometric Methods

**DOI:** 10.3390/foods14071267

**Published:** 2025-04-03

**Authors:** Volodymyr V. Tkach, Tetiana V. Morozova, Isabel O’Neill de Mascarenhas Gaivão, Yana G. Ivanushko, José Inácio Ferrão da Paiva Martins, Ana Novo Barros

**Affiliations:** 1 General and Material Chemistry Department, Chernivtsi National University, Kotrsyubynsky Str. 2, 58000 Chernivtsi, Ukraine; 2 Faculdade de Engenharia, Universidade do Porto, Rua Dr. Roberto Frias, s/n, 4200-065 Porto, Portugal; jipm@fe.up.pt; 3 Ecology and Environmental Protection Department, National Transport University, Omelianovych-Pavlenko Str. 1, 01001 Kyiv, Ukraine; tetiana.morozova@ukr.net; 4 Veterinary and Animal Research Centre (CECAV), University of Trás-os-Montes e Alto Douro (UTAD), 5000-801 Vila Real, Portugal; igaivao@utad.pt; 5 Disaster and Military Medicine Department, Bukovinian State Medical University, Teatralna Sq. 9, 58001 Chernivtsi, Ukraine; yana_iv@ukr.net; 6 Centre for the Research and Technology of Agro-Environmental and Biological Sciences (CITAB), University de Trás-os-Montes e Alto Douro (UTAD), 5000-801 Vila Real, Portugal

**Keywords:** sucralose, food safety, analytical methods, sensing techniques, spectrophotometry, HPLC, electrochemical analysis, environmental impact, degradation products

## Abstract

This review presents an in-depth analysis of the latest methods used for the determination of sucralose (E955), focusing on research conducted over the past 10 years. As a widely used sugar substitute in the food and pharmaceutical industries, sucralose has raised concerns about its environmental persistence, potential genotoxicity, and health impacts. This study examines several spectrophotometric, chromatographic, and electrochemical techniques, evaluating their sensitivity, selectivity, and limitations in differentiating sucralose from natural carbohydrates and other sweeteners. The review highlights the pressing need for novel detection methods that not only improve accuracy in trace detection but also address growing concerns about its bioaccumulation and conversion into harmful metabolites. Advancing these analytical techniques is essential for enhancing food safety, public health surveillance, and environmental risk assessment. Chromatographic methods are dominant in sucralose determination in foods and environmental objects, as they allow the determination of sucralose at micro- and nanomolar levels. However, spectrophotometric and electrochemical methods are frequently used as complementary to chromatographic methodologies, sensitizing them. On the other hand, purely spectrophotometric methods are less popular, and electrochemical methods remain underdeveloped. Therefore, the advancement of sucralose determination must be due to cheaper chromatographic and classical electrochemical methods.

## 1. Introduction

The term “sweetener” [1,2,3,4] generally refers to a substance added to food and drinks with the purpose of sweetening them. Common sugar or sucrose is the most used sweetener in the world. However, the abusive consumption of sugar may lead to complexities like *diabetes mellitus* types I and II, which is the reason why sugar substitutes are widely used as dietetic sweeteners.

**Figure 1:**
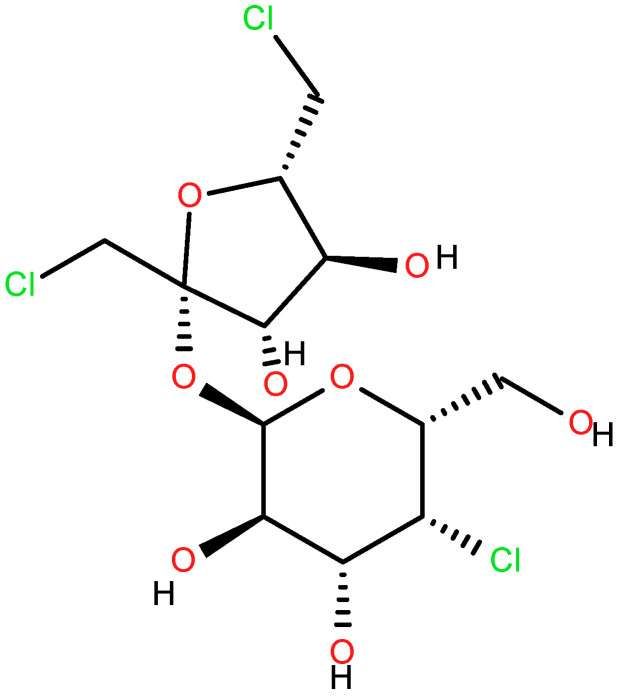
Sucralose.

Sucralose (Figure 1) is one of the dietetic sweeteners most used as a flavor corrector in Portugal and throughout the European Union in the alimentary and pharmaceutical industries [5,6,7,8,9,10], and the ADI level for sucralose is set at 5 mg/kg. In the USA, it is also known as Splenda^®^, the commercial name of the sweetener, in which sucralose is the main component, while dextrose and maltodextrin are present as minor ingredients. In Codex Alimentarius, it is registered as E955. It is a trichloro-substituted derivative of galactosucrose, which has twice the sweetness of saccharin, triple the sweetness of aspartame, and is up to a thousand times as sweet as common sugar. When it comes to physical properties, free sucralose is a white, shiny, odorless substance that is soluble in water. Contrarily to other traditional sweeteners (like aspartame, acesulfame K, saccharin and stevia), it is a carbohydrate derivative, which is the reason why it tastes extremely sweet.

**Figure 2:**
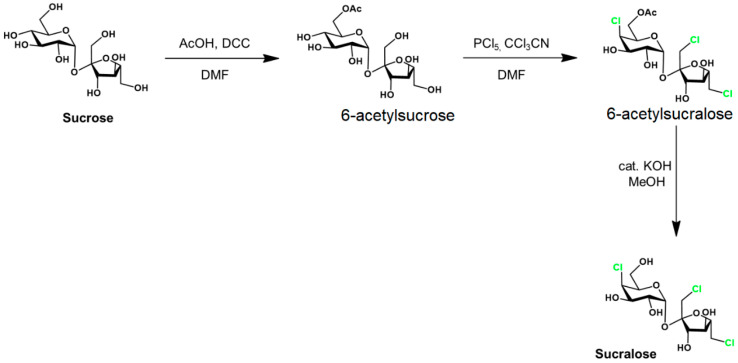
Sucralose synthesis from sucrose. Adapted from [6] with the correspondent’s permission.

Sucralose is synthetized in the food industry from common sugar via several steps (Figure 2).

The acylation in the first stage may also be facilitated by diethylazodicarboxylate. The C4 epimerization is realized in the second stage. This synthesis involves either the toxic reagents (DCC, DAAD, and PCl_5_) or the intermediate (6-acetyl sucralose), as mentioned below.

Despite being considered safe for use by people with diabetes and athletes, its harmful effects on human health and the environment are still unknown, and some of its negative effects have only begun to be studied now. A recent study involving pregnant and breastfeeding women [11,12,13] confirmed that sucralose enters breast milk, causing irreparable damage to the development of the gut microbiota of the human fetus in the last months of pregnancy, as well as in neonates and babies—this is the reason why its safety for use during pregnancy and breastfeeding is still questioned.

**Figure 3:**
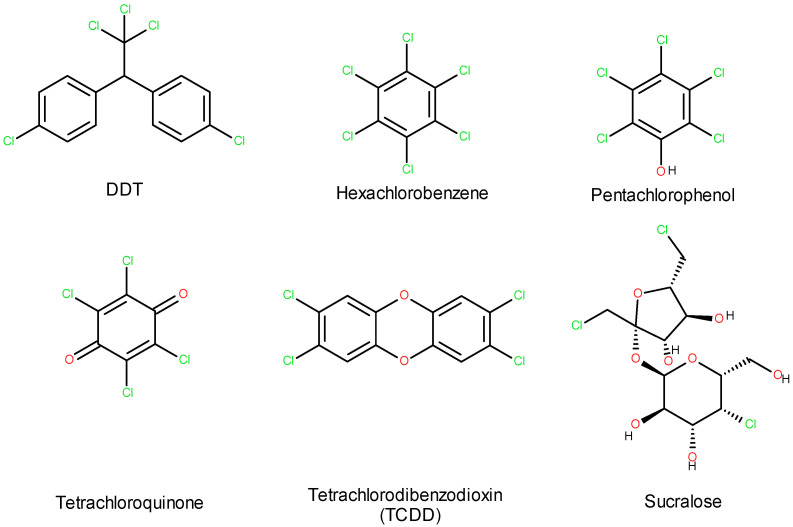
Sucralose among organic halogen compounds.

Moreover, due to its low biodegradability, sucralose tends to persist and accumulate in the environment [14,15,16]. Furthermore, when sucralose decomposes thermally or by some bacteria, it transforms into toxic compounds such as dioxins and tetrachlorodibenzofurans. Sucralose belongs to the group of halogenated organic compounds (Figure 3), which are known for their potential short- and long-term ecotoxic effects.

Another critical issue yet to be addressed about this compound is the presence of 6-acetyl sucralose, the industrial precursor of the sweetener (Figure 2), in reasonable concentrations in industrial product samples. Furthermore, its appearance in the human intestine is also likely, where it is precisely esterified by the hydroxyl linked to the C6 carbon atom. Recent research has proven both the genotoxicity of 6-acetyl sucralose [17,18,19,20] and the formation of acetylated metabolites in significant concentrations in the human body [21,22].

Toxicological studies of the steric derivative of sucralose proved that the mechanism of its genotoxicity can be considered clastogenic (initiator of breaks in the DNA structure) [23,24,25,26,27,28,29]. Even microscopic concentrations of 6-acetyl sucralose, which can be detected in industrial samples and beverages, exceed the safe threshold of 0.15 μg/person/day. The level of 6-acetyl sucralose ester increased in intestinal epithelial cells, expressing genes linked to inflammation, oxidative stress, and carcinogenesis, including *MT1G* and *SHMT2* [23,24,25].

Another detrimental effect of the steric derivative is its inhibition of CYP1A2 and CYP2C19, enzymes from the cytochrome P450 family that play a crucial role in converting various food substances into more bioavailable forms. This inhibition can lead to secondary toxic effects [26,27,28,29]. The increased genotoxicity of 6-acetyl sucralose compared to sucralose is attributed to the heightened reactivity of the secondary organic chloride attached to the C4 carbon atom, which is further activated by the electron-withdrawing effect of the steric group. This same activation is also responsible for the compound’s mutagenic potential. A comprehensive review [30] explores the toxicological effects of sucralose in detail. The assessment of industrial production and the environmental fate of sucralose is becoming increasingly important, particularly due to its potential use as a tracer of anthropogenic activity, presence, and pollution [31,32,33,34,35,36,37,38,39,40]. In [31], the first cradle-to-factory-gate life cycle assessment (LCA) of sucralose production from cane sucrose in the USA was presented. The study estimated the global warming potential of producing 1 kg of sucralose at 71.82 kg CO_2_-eq/kg, primarily due to the high consumption of ecotoxic compounds during synthesis.

In [32], the neurobehavioral and cardiotoxic effects of acesulfame and sucralose were examined in *Daphnia magna*, revealing that while acesulfame exhibited greater toxicity, both substances caused dose-dependent physiological and behavioral impacts, as well as changes in acetylcholinesterase (AChE) activity. These findings highlight the need to reassess risk assessment thresholds in wastewater.

Young et al. [33] measured sucralose concentrations in wetlands, reporting values of 26 ± 2 ppb in surface water and 20 ± 6 ppb in outfall samples. Their results indicated that sucralose was neither adsorbed nor chemically altered by the wetland environment, reinforcing its stability and persistence [14]. Similarly, in [34], predicted levels of sucralose in actual water reuse scenarios were found to be comparable to those of highly stable per- and polyfluoroalkyl substances (PFAS), further supporting its potential classification as a persistent halogenated organic compound.

Sucralose has also been used as a tracer of anthropogenic activity. In [35], both sucralose and caffeine were detected in coral reef ecosystems, demonstrating their entry into the food chain of Vatia Bay, American Samoa, through wastewater discharge from local villages. Likewise, in [36], these compounds were identified as indicators of domestic wastewater contamination in the Laurentian Great Lakes basin.

The impact of sucralose on microbial communities has also been explored. In [37], sucralose exposure was shown to reduce microbial activity in marsh environments, with a dose-dependent decrease in diatom activity and an increase in cyanobacterial activity, suggesting shifts in ecosystem dynamics. Furthermore, in [38], sucralose was found to be the most persistent among four artificial sweeteners (acesulfame K, cyclamate, saccharin, and sucralose), undergoing minimal degradation during water treatment processes. While short-term ecotoxicity appears to be low, the long-term environmental effects remain under investigation.

Given these concerns, the development of efficient, rapid, and sensitive methods for sucralose detection in food, beverages, and environmental matrices is both timely and essential [39,40].

In recent years, both the EU and USA have seen noticeable shifts in dietary trends, particularly in relation to the consumption of bioactive compounds. The European market has been increasingly focused on healthier lifestyles, with a growing demand for functional foods and beverages that offer nutritional benefits beyond basic nourishment. This has led to a rise in the consumption of products rich in bioactive compounds, such as polyphenols, flavonoids, and vitamins [41,42,43]. According to recent reports, the EU’s market for functional foods has steadily expanded, driven by consumer interest in prevention and well-being [44,45,46].

Similarly, in the USA, there has been an increased awareness of the health benefits associated with bioactive compounds, contributing to a higher per capita intake. The “dose per capita” of specific bioactive compounds, such as antioxidants from fruits and vegetables, has seen an upward trend, as individuals seek to improve their health through diet. This shift is supported by the increasing availability of bioactive-enriched foods in the market, as well as by scientific studies that emphasize their role in disease prevention and overall health promotion [47,48,49].

Including these consumption trends helps to underscore the relevance of bioactive compounds in both the EU and USA, and their potential impact on public health [50,51,52].

## 2. Materials and Methods

The selection of articles for this review followed a structured and transparent approach. Relevant studies were identified through a comprehensive search in databases such as Web of Science, Scopus, and PubMed, using specific keywords related to chromatographic, electrochemical, and spectrophotometric methods for sucralose determination.

Inclusion criteria were based on the relevance of the studies to the topic, methodological quality, and publication in peer-reviewed journals. Additional references were identified through citation analysis to ensure a comprehensive overview of the field. After an initial screening of titles and abstracts, full texts were assessed to confirm their suitability for the review.

This selection process ensured the inclusion of high-quality studies that contribute to understanding the advancements and challenges in sucralose determination.

## 3. Sucralose Determination Methods

### 3.1. Spectrophotometric Methods

Considering the chemical composition of sucralose, the presence of chlorine atoms directly bonded to the carbohydrate moiety plays a crucial role in its efficient immobilization and selective detection. This occurs due to the electronic effects of chlorine and the unique quantum-chemical properties of the C–Cl bond, which contribute to the differentiation of sucralose from other similar compounds [53,54]. Additionally, the reactivity of the C–Cl bond can be effectively utilized for the derivatization of sucralose, enhancing its visibility in the UV/Vis spectrum. Various spectral methods have been employed for the quantification of sucralose, both independently and in combination with chromatographic techniques, which remain the most prevalent approaches for sucralose determination. These methods were discussed in detail in Section 3.2, where a comprehensive review of spectral techniques for sucralose analysis was presented. In study [55], the photodegradation of sucralose in an alkaline medium (pH = 12) was investigated. The process involved an initial hydrolysis step, followed by oxidation to a carbonyl derivative that exhibited photoactivity at 270 nm, eventually further oxidizing into a carboxylic acid. The detection limit for this method was determined to be 0.02 g/L, and the calibration curve followed Beer’s law. The method was tested on commercial sweetener samples and beverages, with its effectiveness confirmed by the findings in [56]. Additionally, it was demonstrated that other common components in commercial sweeteners, such as binders and maltodextrin, did not significantly affect the method’s efficiency.

In study [57], two spectrophotometric kinetic methods were developed for the determination of sucralose in tablets, where it was used as a flavor corrector. Both methods were indirect, relying on the oxidation of sucralose by permanganate in an alkaline medium (610 nm) and by cerium ammonium sulfate in acidic solutions (320 nm) under thermostated conditions at 60 °C. The calibration curves for these methods were linear within the ranges of 4–16 μg/mL and 10–30 μg/mL, respectively, indicating that while both approaches were sensitive, the first method demonstrated higher sensitivity for sucralose detection in pharmaceuticals. However, the application of these methods to food products may be less reliable due to the presence of additional compounds that can also be oxidized by tetravalent cerium and heptavalent manganese, potentially leading to interference. Study [58] explored an FTIR spectroscopic method combined with a machine learning tool for the determination of sucralose, sodium cyclamate, sodium aspartame, sodium saccharin, and aspartame. Multivariate models were developed, tested, and compared with the HPLC reference method, showing good agreement between the two approaches. However, the method was found to be less precise in random forest and well water samples than in previously prepared solutions, highlighting the need for further training of the model. In study [59], the influence of sucralose on ovalbumin non-covalent interactions was investigated using IR spectrometry. A comparison of IR spectrum intensities revealed that ovalbumin began to denature at different pH levels in the presence of sucralose, suggesting that sucralose may contribute to protein denaturation and degradation. Study [60] focused on the determination of natural carbohydrates (sucrose, fructose, galactose, and glucose) and five sweeteners (acesulfame K, neotame, saccharin, rebaudioside A, and sucralose) using ATR-FTIR, NIR, and Raman spectroscopy. The ATR-FTIR spectrum of sucralose exhibited characteristic bands at 618 cm^−1^ (C–Cl stretch), 642 cm^−1^ (C–Cl stretch), 857 cm^−1^ (C–H bending), 890 cm^−1^ (C–H bending), 1001 cm^−1^ (C–O stretch), 1030 cm^−1^ (C–O stretch), 1092 cm^−1^ (C–O stretch), and 3457 cm^−1^ (O–H stretch). The NIR bands corresponded to 4046 cm^−1^ (CH combinations), 4413 cm^−1^ (CH_2_ combinations), 5766 cm^−1^ (CH and CH_2_ first overtone), and 5848 cm^−1^ (CH and CH_2_ first overtone combinations). Raman spectroscopy identified additional sucralose bands at 353, 536, 624, 664, 710, 745, and 775 cm^−1^, corresponding to various vibrational modes. These spectral profiles provide valuable reference data for the identification and quantification of sugars and sweeteners. Study [61] investigated the thermal degradation products of isolated sucralose using FTIR and HRMS, confirming the formation of highly toxic polychloroarenes during sucralose dehydration. Other compounds, including CO_2_ and HCl, were also identified, reinforcing concerns about the production of hazardous substances when sucralose is exposed to high temperatures, such as during baking.

In study [62], sucralose was detected in e-liquids using AMS and NIR spectroscopy. The concentration measurements were validated by HPLC/MS, and the method demonstrated the advantage of analyzing e-liquids in their original state without requiring sample preparation. The matrix-matched calibration set enabled the detection of sucralose at levels below 0.2%.

Study [63] employed UV/Vis spectroscopy to detect sucralose, both with and without HPLC. The derivatization of sucralose with *p*-nitrobenzoyl chloride resulted in an adduct with strong absorption at 260 nm, enabling detection at the micromolar level. Additionally, refractive index and mass spectrometry detectors were also used for sucralose analysis [64,65].

The general and succinct overview of the spectrophotometric determination of sucralose in the presence of different analytes is given in Table 1.

From this brief review of purely spectroscopic methods for sucralose quantification, it can be concluded that while some require additional steps, such as derivatization in UV/Vis spectroscopy, they are capable of detecting the sweetener at micromolar concentrations, achieving nearly 100% recovery in some cases. This makes them a solid foundation for chromatographic methods, which will be analyzed in the next section.

### 3.2. Chromatographic Methods

Chromatographic methods are very popular in food analysis, due to their high precision, wide range of analytes, and low sample volumes [66,67]. For this reason, chromatographic methods are widely used in food analysis and may be applied for sucralose determination in different media: commercial samples; food samples (including beverages, sport food, and dietetic food); and environmental samples.

Among other chromatographic methods, those applied solely and in combination with spectral (see Section 3.1) and electrochemical (see Section 3.3) methods are the most popular for sucralose quantitative determination. This subsection is dedicated to the review of the state of the art in chromatographic methodologies applied to sucralose determination.

Gas chromatography methods are frequently used in sucralose determination, due to their high sensitivity. For example, in study [68], gas chromatography, combined with mass spectrometry, was used to detect sucralose in commercial Splenda^®^ samples from Sigma (St Louis, MO, USA). Sucralose was derivatized as trimethylsylyl ether and myo-inositol was used as an internal standard. The amount–response dependence for SucrTMS was observed to be rectilinear in the range of 0.005–0.06 mg/mL, with 250 pg being the low detection limit. An analogous process was developed in [56], in which sucralose was detected by GC/MS in wastewater, etherifying all five sucralose hydroxyls with TMS, using deuterated internal standard, and it was capable of detecting at least 21.8 ng/L of sucralose with nearly 0.1% wastewater contribution.

A CGGC-based method has been developed in [69] by Farhadi et al., for the determination of sucralose alone and in the presence of lactulose and mannitol in urine. The same method was used to verify the possibility of metabolism of all three sugar substitutes by intestinal bacteria. It was confirmed that only lactulose was metabolized, whereas sucralose and mannitol were not. The variation coefficient decreased nearly four times and the sensitivity grew by 200–2000 in comparison to PCGC. The linear range for sucralose was between 0.2 and 40 g/L.

Liquid chromatography and its derived methods are widely used and referenced for the determination of sucralose [70,71,72,73,74,75,76,77,78,79,80,81,82,83,84,85,86,87,88,89,90,91,92,93,94,95,96,97,98,99,100]. These methods can be employed either alone or combined with spectral and electrochemical techniques, taking advantage of both. In combined methods, the disadvantages of one technique are compensated by the advantages of another.

For example, in [70], Greibe et al. describe an LC-MS/MS-based method for quantifying acesulfame K, cyclamate, saccharin, and sucralose in human plasma, amniotic fluid, and breast milk. Linear functions were established for each of the sweeteners analyzed. Accuracy, precision, long-term stability, and freeze–thaw tests met the validation criteria, with the most efficient recovery of sucralose from amniotic fluid, followed by plasma.

In [71], two quadrupole mass techniques were used to detect sucralose in water. Depending on the solution’s pH, sucralose was detected either in the deprotonated form or as a sodium adduct. Both LC/MS-MS and LC/Q-TOF-MS were efficient in determining sucralose, with LC/MS-MS showing higher sensitivity in positive-ion mode, with detection limits of 15 ng/L.

In [72], acesulfame K, aspartame, cyclamate, dulcin, glycyrrhizic acid, neotame, neohesperidin dihydrochalcone, saccharin, sucralose, and stevioside were detected by LC/TMS in alcoholic and non-alcoholic beverages. The detection limit ranged from 10 to 500 pg/L, and the sensitivity of the method depended on the pH of the food matrix in which the concentration was measured.

The work in [73] was dedicated to the LC/TMS analysis of sucralose in the presence of five natural sweeteners—mogroside V, neohesperidin dihydrochalcone, rebaudioside A, stevioside, and glycyrrhizic acid. The detection limit was around 10 pg/L, which is very low. The molecular adduct with cesium cation was used for MS measurements, providing high sensitivity and signal-to-noise ratio. Thus, the sensor may be excellent for measuring small concentrations of sweeteners.

Loos et al. [74] proposed a notable analytical method for monitoring sucralose in European river waters. Their LC-MS/MS measurements, based on solid-phase extraction (SPE) and electro-spray ionization (ESA) in neutral and mildly acidic media, had the objective to measure sucralose concentration in river waters as a tracer of anthropogenic activity. The sucralose analysis confirmed the presence of sucralose in aquatic environments at concentrations reaching up to 1 μg/L, particularly in Western European countries.

Batchu et al. [75] developed an SPE-LC-MS/MS-based method for detecting sucralose in rivers across West Florida. The detection limits ranged from 8.5 ng to 2.5 μg/L, with sucralose frequently detected at concentrations as high as 18 μg/L. Recovery rates varied between 85% and 113%, suggesting that sucralose consumption in the US via drinking water sources is estimated at approximately 5.0 mg per person per day—twice as high as in the European Union. Notably, this study also identified a photolysis product of sucralose for the first time. High-performance liquid chromatography (HPLC) remains one of the most widely used techniques for determining sucralose in various matrices, including food products, pharmaceuticals, and environmental samples. These methods provide reliable detection and often serve as reference techniques for alternative analytical approaches, such as purely spectroscopic or electrochemical methods [76,77].

Johns and Dowlati [78] developed an HPLC-UV method at 192 nm for the simultaneous determination of sucralose and acesulfame in OEMS. This technique ensured effective separation and quantitative analysis, with no peak interference between the analytes [55]. Yan et al. [79] successfully applied HPLC-ELSD to determine sucralose in the presence of sugars and other sweeteners. Their method achieved complete separation in a 14 min single run on a C18 column, demonstrating good linearity, a low detection limit (0.5–2 μg/mL), and high repeatability. Wu et al. [80] described an ultra-high-performance liquid chromatography tandem mass spectrometry (UHPLC/TMS) method for nanomolar detection of sucralose and acesulfame K in well water from Alberta, Canada. Detection limits were as low as 200 pcg/L for acesulfame K and 5 ng/L for sucralose, with both sweeteners frequently detected in domestic wells, underscoring the need for ongoing water quality monitoring. Ma et al. [81] developed an ultrasensitive UHPLC-PDA-CAD method for detecting sucralose in Chinese spirits alongside nine other sweeteners. Their approach, which utilized a highly sensitive photoaerosol detector, achieved a limit of detection (LOD) of 0.18 μg/g. This method was later employed in China’s nationwide initiative, the “Special Action Against Counterfeit and Shoddy White Spirits” [82,83].

Due to its higher electronegativity compared to sucrose, sucralose is particularly well suited for detection via ion-exchange chromatography. In [84], it was readily identified without requiring any sample preparation using high-performance anion-exchange chromatography (HPAE) coupled with pulsed amperometric detection. This technique exhibited a broad linear range spanning three orders of magnitude, making it an efficient approach for sucralose quantification. A similar method was described in [85].

In [86], the photodegradation of sucralose was investigated using the photo-Fenton process, with monitoring carried out via HPLC analysis. The process, photocatalyzed by TiO_2_, achieved an almost complete mineralization of sucralose over a five-hour reaction period. However, natural components of Splenda^®^, such as maltodextrin and dextrose, were not fully degraded. Despite the high efficiency of sucralose removal through Fenton processes, the method may not be entirely environmentally friendly due to the presence of organic chlorine in sucralose. This chlorine, which was not accounted for by the authors of [86], oxidizes into gaseous chlorine or chlorine oxides—compounds that, although potentially reusable (e.g., ClO_2_), remain ecotoxic and may pose short-term environmental risks greater than those of sucralose itself.

Sarotra et al. [87] developed an HPLC-ELSD-based method to determine sucralose in the presence of sucrose, mannitol, and lactulose, aimed at estimating intestinal permeability in patients with active ulcerative colitis. The method achieved an analytical recovery rate of 95–146% in urine samples, with a detection limit of 50.9 mg/L and a quantification limit of 170.7 mg/L—offering a highly effective, non-invasive approach for gut permeability assessment.

Mabrouk et al. [88] introduced an HPLC-ELSD method for the simultaneous determination of sucralose and α-lipoic acid in bulk and pharmaceutical dosage forms. Chromatographic separation was performed using a C18 (5 µm) column with two mobile phases at pH 2.5 (for ALA) and pH 7.0 (for sucralose). A previously established HPLC-UV method [89] served as a reference, and the new method demonstrated slightly higher mean recovery rates, suggesting improved precision over the reference technique.

Morlock et al. [89,90] developed an HPLC-UV method for sucralose determination, employing derivatization with compounds such as p-aminobenzoic acid, aniline, and diphenylamine orthophosphoric acid to enhance UV absorption. This approach reduced the quantification limit to 100 ng/L, with a recovery rate of 80%. The method, which required less than five minutes per sample and allowed the simultaneous analysis of up to 17 samples, showed strong concordance with results obtained through HPLC-MS.

Another efficient sucralose quantification approach, based on solid-phase extraction and HPLC-MS/MS, was referenced in [91]. This method provided a linearity range of 10–500 ng/mL, with recovery rates reaching 110.3%. The limit of detection for sucralose was 0.02 mg/kg in spirits and 0.1 mg/kg in other samples.

An HPLC-based method was also developed for monitoring sucralose concentrations in cookies [92]. A total of 80 biscuit samples, randomly selected from the market, were analyzed, yielding recovery rates of up to 100% and a detection limit of 2 mg/kg. The results confirmed that the sucralose content in products labeled as containing the sweetener complied with Chinese regulatory limits (0.25 g/kg). The method proved suitable for use by food inspection authorities.

Sousa et al. [93] proposed an HPLC-ELSD method for quantifying acesulfame-K, aspartame, cyclamic acid, neotame, saccharin, and sucralose in beverages using a dilute-and-shoot extraction approach. This method reduced solvent consumption and enabled analysis within 35 min. A study of 64 non-alcoholic beverages found no undeclared sweeteners in 19 samples. However, aspartame was detected at concentrations exceeding declared levels in carbonated soft drinks and powdered beverages, while sucralose was identified only in sports drinks (1.66 mg/100 mL). Sweetener concentrations varied between 54.1% and 194% of the declared amounts.

Hellwig [94] conducted HPLC-TOP-MS monitoring of sucralose dehydration, confirming that either the galactopyranose or fructofuranose moiety underwent dehydration, leading to the formation of chlorinated organic compounds—each more hazardous than sucralose itself. When dehydration occurred in the presence of protein, 3-chlorotyrosine was formed. The direction of sucralose degradation depended on the composition of the initial food sample, including its pH. Additionally, heating in the presence of sucralose increased the concentration of 5-hydroxymethylfurfural (5-HMF), suggesting that 5-HMF is also a sucralose degradation byproduct. The presence of chlorofurans and chlorofurons—known for their environmental toxicity and potential mutagenicity—was confirmed.

Soyseven et al. [95] reported an HPLC-ELSD method for quantifying sucralose in soft drinks and candies. The limits of detection (LOD) and quantification (LOQ) ranged from 1.96 µg/mL and 6.53 µg/mL, respectively, making the method suitable for food product analysis.

A critical analysis of sucralose and acesulfame K as water contaminants was presented in [96], accompanied by LC-MS analysis of surface and groundwater from the Danube and Sava Rivers near Belgrade, Serbia. Both sweeteners were widely detected, with sucralose concentrations reaching up to 4.756 ng/L. Although these levels did not pose immediate risks to aquatic life, seasonal variations, particularly in summer when river usage increases, could lead to higher concentrations. Given sucralose’s low biodegradation rate, its presence is expected to persist and accumulate in both river and seawater, particularly in the Black Sea.

Naik et al. [97] developed an HPLC and TLC method for quantifying acesulfame K, sucralose, saccharin, and aspartame in food, beverages, and chewing gum, aiming to support regulatory agencies and public awareness regarding artificial sweetener content. Sucralose concentrations ranged from 83 to 93 mg/100 mL in beverages, and from 82 to 155 mg/100 g in chewing gum, all within the limits set by national regulations [98,99,100].

In [101], Martínez et al. monitored the presence of sucralose alongside other ecotoxic compounds in the surface and tap water throughout El Salvador by using LC-MS/MS with a detection limit of 94 ng/L. The concentration measurement showed that the concentration of sucralose at dry season is higher than in wet season, reaching the values round 2 μg/L, which indicates its high environmental stability. This allowed the use of the concentration measurement of sucralose to trace the anthropogenic activity.

Covic et al. [102] explored the potential effects of sucralose on insulin-signaling pathways. Cells exposed to sucralose, both alone and in combination with L-dopa and insulin, exhibited alterations in insulin response mechanisms, with lipid composition changes confirmed through HPLC analysis.

Nicoluci et al. [103] developed a UHPLC-MS/MS method for detecting and quantifying sucralose in the presence of acesulfame K, aspartame, advantame, sodium cyclamate, neotame, saccharin, stevioside, and rebaudioside A in 42 commercially available Brazilian products. Recovery rates ranged from 89% at high concentrations to 200% at low concentrations. No undeclared sweeteners were detected, contrasting with findings from a previous study [104] that reported the presence of five undeclared sweeteners.

Agulló et al. [105] demonstrated that the presence of non-sugar sweeteners, such as sucralose and stevia, enhanced polyphenol bioavailability compared to sucrose. Polyphenolic composition and sweetener content were analyzed via HPLC-DAD/MS. While the study suggested sucralose and stevia as alternatives to sucrose, it did not address potential toxic or ecotoxic effects, nor the negative influence of steviol on fertility.

In [106], QuEChERS rapid pretreatment combined with HPLC-DR was used to quantify sucralose in soy sauce produced in China. The method exhibited linearity in the 20–1000 mg/L range, with recovery rates between 84.2% and 93.4%. The sucralose concentrations in analyzed samples complied with Chinese national regulations.

BeiBei et al. [107] presented an SPE-HPLC-ELSD method for quantifying sucralose in deproteinized canned food samples. The method exhibited a linear range between 20.26 and 405.2 μg/mL, with average recoveries varying from 99.6% to 105.3%. Its accuracy and sensitivity make it an efficient tool for sucralose determination in canned food.

Huang et al. [108] investigated sucralose biodegradation using UPLC-QTOF-MS to analyze the process carried out by an enriched bacterial consortium. Their findings confirmed total sucralose degradation within four days. However, the degradation products were found to be potentially more ecotoxic and mutagenic than sucralose itself, primarily due to incomplete dichlorination by the bacterial consortium. Further dehydration of these byproducts could lead to even more environmentally aggressive compounds. This highlights the ongoing challenge of ensuring both safe sucralose biodegradation and its potential reuse.

A succinct overview of the chromatographic methods for sucralose determination is given in Table 2.

Considering these findings, chromatographic methods offer ultrasensitive detection of sucralose across various media. Their combination with other techniques, such as spectrophotometric or electrochemical methods, enhances their efficiency and broadens their applicability. These methods enable effective monitoring of sucralose not only in food, beverages, e-liquids, and chewing gum but also in environmental contexts, tracking its presence in ecosystems such as seas, rivers, marshes, and wetlands. Furthermore, they help assess bioaccessibility and biodegradation potential. Additionally, chromatographic analyses provide insights into the mechanisms of sucralose thermal dehydration, revealing the formation of various halogenated organic byproducts known for their toxicity.

### 3.3. Electrophoretic and Electrochemical Methods

As mentioned in the previous subsection, chromatographic methods are the most widely used for sucralose quantification. However, they often require expensive equipment, as well as intricate and time-consuming sample preparation, and lack portability, limiting their applicability for field measurements of sucralose concentrations. Given these limitations, electrochemical and electrophoretic methods present a promising alternative to HPLC-based techniques or can be effectively integrated with them. Furthermore, chromatographic analyses contribute to understanding sucralose’s environmental fate in seas, rivers, marshes, and wetlands, aiding in the assessment of its bioaccessibility and biodegradation potential. Additionally, these methods provide insights into the mechanisms of sucralose thermal dehydration, revealing the formation of various halogenated organic byproducts known for their toxicity. However, electrophoretic and electrochemical methods are less commonly employed for sucralose determination, despite the molecule’s electrochemical activity due to its hydroxyl groups [109,110]. This limited use can be attributed to challenges such as difficulty in achieving proper peak separation, which often requires specialized electrode modifications. Additionally, factors like sample matrix composition, interference from other substances, and the need for optimized buffer systems can affect the sensitivity and resolution of these methods, contributing to their slower development and adoption in sucralose analysis [111,112].

In [113], Stojka et al. developed a capillary electrophoretic method for sucralose determination in a 3,5-dinitrobenzoic acid buffer at pH 12.1. The detection and determination limits were observed at 28 and 42 mg/L, with a repeatability of 4.2% for the signal area, making it suitable for detecting sucralose in food.

McCourt et al. [114] combined capillary electrophoresis with indirect UV screening using the same buffer, achieving detection of sucralose in beverages, yogurts, and candy with a limit of more than 30 mg/kg, and a linearity observed between 50 and 500 mg/kg. However, sample preparation was complex. A similar process was conducted by Nikolelis et al. [115], using a biosensor based on a bilayer lipid membrane, with a detection limit in the same range.

Ayyappa et al. [116] developed a method for the electrophoretic sucralose determination using amines as background electrolyte. The electrophoretic analysis, coupled with UV detection, had been previously supported by DFT calculations and was realized via selective derivatization of sucralose via a nucleophilic substitution reaction at pH = 12, maintained by morpholine buffer. This is efficient for sucralose determination in the presence of natural carbohydrates, as sucralose reacts specifically by chlorine atoms, whereas the carbohydrates manifest it with greater difficulty. The linearity, studied in the range of 2–10 mmol/L, had a correlation coefficient of 0.9942. The recoveries ranged from 94 to 98%.

The first classical electrochemical determination of sucralose, which had been theoretically proposed in [117], was achieved in [118] using an electrochemical biosensor based on a ZnONPS/GO-based electrode with immobilized laccase. The electrooxidation of sucralose was driven by proton loss from the unique exocyclic hydroxymethyl group in a mildly acidic medium. The linearity range was observed between 0.025 and 0.1 mM, with a detection limit of nearly 320 nmol.

In [119], sucralose was detected in soft drinks using cyclic voltammetry (CV) with two oxidation peaks observed at −0.75 V and +1 V at a bare glassy carbon electrode (GCE). Sucralose detection allowed for the quantification of sweetener concentration and the calculation of safe daily drinking doses in terms of sucralose consumption. However, electrochemical determination on a bare electrode is not ideal for detection, so electrode modification becomes essential.

### 3.4. Comparative Overview of Chromatographic and Electrochemical Approaches for Sucralose Determination

Based on a brief overview of spectrophotometric, chromatographic, and electrochemical methods, it can be concluded that chromatographic techniques are the most widely used, sensitive, and frequently employed for sucralose determination. These methods offer very high sensitivity and are capable of detecting sucralose concentrations in the nanomolar and even picomolar range, even in the presence of other sweeteners and natural carbohydrates. However, they typically combine chromatography with other techniques, such as spectroscopic and electrochemical methods, and require slow, costly equipment.

In contrast, direct electrochemical methods, which could offer a quicker and more sensitive alternative, are still less commonly used. This is primarily due to the need for electrode modification for proper peak separation. Therefore, the development of direct electrochemical methods and their integration with HPLC appears to be a promising approach for sucralose determination in food, beverages, pharmaceuticals, and environmental samples.

As a result, future research on sucralose determination in food should focus on improving and reducing the costs of HPLC methods while also developing simple, portable, rapid, and precise direct electrochemical methods.

## 4. Conclusions

A review of the sucralose detection methods developed over the past 25 years highlights the continued dominance of chromatographic techniques due to their unparalleled sensitivity and precision in quantification. Despite this, these methods often require costly equipment, involve complex procedures, and are time-consuming, which limits their accessibility and practicality in routine analysis. In contrast, electrochemical methods, which benefit from the electrochemical activity of sucralose, offer the potential for faster, more cost-effective detection. However, direct electrochemical detection techniques, which could serve as a promising alternative without sacrificing sensitivity, have not yet been fully developed or optimized.

To address these challenges, future research should prioritize the development of direct electrochemical sensors for sucralose detection, focusing on improving their sensitivity, selectivity, and practicality for real-world applications. Additionally, further optimization of high-performance liquid chromatography (HPLC) methods is necessary to reduce costs and simplify the process, making it more accessible for widespread use. Combining chromatographic techniques with electrochemical and spectroscopic detection could provide a more holistic approach, capitalizing on the strengths of each method and creating a versatile, multi-dimensional platform for accurate and rapid analysis.

This integrated approach would not only improve the overall efficiency and cost-effectiveness of sucralose analysis but also facilitate its detection across a wide range of food, pharmaceutical, and environmental samples. Ultimately, such advancements in analytical methodologies will be crucial for ensuring food safety, public health monitoring, and environmental risk assessment, especially as concerns about the environmental impact and potential health risks of sucralose continue to grow. The development of these more efficient and accessible detection techniques will play a key role in advancing scientific knowledge and addressing public and regulatory concerns regarding sucralose.

## Figures and Tables

**Table 1:** Overview of spectrophotometric determination of sucralose.

Presence of Analytes	Objects	Methodology	Concentrations	Reference
Sucralose, its degradation products, maltodextrin, and binders	Commercial sucralose samples which have undergone photodegradation	UV/Vis	Over 0.2 g/L	[55]
Sucralose	Pharmaceutical formulations	UV/Vis kinetic determination	4–16 μg/mL	[57]
			10–30 μg/mL	
Sucralose, sodium cyclamate, sodium aspartame, sodium saccharin, and aspartame	Beverages (diet teas)	FTIR	NQ	[58]
Sucralose and ovalbumin degradation products	Chicken egg ovalbumin samples	IR	NQ	[58]
Sucrose, fructose, galactose, glucose, acesulfame K, neotame, saccharin, rebaudioside A, and sucralose	Commercial sweetener samples	ATR, FTIR, NIR, Raman	(Qualitative analysis) NQ	[60]
Sucralose alongside its thermal degradation products	Commercial sweetener samples in normal and heated form	FTIR, HRMS	NQ	[61]
Sucralose	Electronic liquids	AMS, NIR	0.02–0.22% *w*/*v*	[62]
Sucralose, meso-erythritol, xylitol, d-glucitol, d-mannitol, maltitol, and parachinit	Commercial sweetener samples and dietetic beverages	UV/Vis	10–250 μg/mL	[63]
Sucralose	Liquid and solid alimentary products	UV/Vis, refractometry, MS	1–50 μg/mL	[64,65]

**Table 2:** Overview of chromatographic determination of sucralose.

Analytes	Objects	Methodology	Concentrations	Reference
Sucralose	Splenda commercial sweetener	GC-MS	0.005–0.06 mg/mL	[67]
Sucralose	Well water	GC-MS	Over 21.8 ng/L	[68]
Sucralose, lactulose, mannitol	Urine	GC-MS	0.2–40 g/L	
Acesulfame K, cyclamate, saccharin, sucralose	Biological liquids	LC-MS/MS	10–500 ng/L	[70]
Sucralose (deprotonated and sodium adduct)	Drinking and wastewater	LC/Q-TOF-MS LC-MS/MS	Over 15 ng/L	[71]
Acesulfame K, aspartame, cyclamate, dulcin, glycyrrhizic acid, neotame, neohesperidin dihydrochalcone, saccharin, sucralose, stevioside	Beverages	LC-MS/MS	10–500 ng/L	[72]
Sucralose, mogroside V, neohesperidin, dihydrochalcone, rebaudioside A, stevioside, glycyrrhyzic acid	Foods, beverages, and dietary supplements	LC-MS/MS	Over 10 ng/mL	[73]
Sucralose	River water	SPE, ESI, Triple/quadruple LC-MS/MS	Over 10 ng/mL	[74]
Sucralose	River water	SPE-LC-MS/MS	8.5–2500 ng/L	[75]
Acesulfame K, aspartame, cyclamate Na, saccharin, stevioside, sucralose	Wine	LC/MS	Over 0.022 mg/L	[76]
Erythritol, xylitol, sorbitol, maltitol, acesulfame-K, saccharin-Na, sucralose, aspartame, cyclamate, alitame, NHDC, advantame, stevioside, neotame, perillartine	Diabetic foods	HPLC/MS/MS	Over 260 ng/L	[77]
Acesulfame-K, sucralose	Foods and beverages	LC-UV	Over 32 mg/L	[78]
Sucralose and 6-acetylsucralose among 9 chlorinated carbohydrates	Foods, beverages, and commercial samples	HPLC-ELSD, LC-MS, NMR	6–600 μg/mL	[79]
Sucralose, aspartame	Well water	HPLC-MS/MS	Over 5 ng/L for sucralose	[80]
Acesulfame, alitame, aspartame, dulcin, neotame, neohesperidine dihydrochalcone, saccharin, sodium cyclamate, sucralose	Chinese white spirits	UHPLC/PDA/CAD	0.5–50 μg/g	[81]
Acesulfame K, saccharin, sucralose, aspartame, steviol glycosides, benzoic acid, sorbic acid	Alcoholic and soft beverages	HPLC-DAD-ELSD	Over 31.4 mg/L	[82]
Sucralose, dextrose, maltodextrin	Splenda^®^, beverages	HPAE, PAD	0.01–40 μM	[84]
Sucralose	Beverages	HPLC, RID	20–400 mg/L	[85]
Sucralose among degradation products, dextrose, maltodextrin	Splenda^®^, sugar-free beverages	HPLC/MS	NQ	[86]
Sugar, lactulose, sucralose, mannitol	Human urine	HPLC	Over 50.908 mg/L for sucralose	[87]
Lipoic acid and sucralose	Pharmaceutical formulations, bulk	HPLC/ELSD	Over 0.320 ppm	[88]
Sucralose	Dietetic products	HPTLC/UV	Over 4 ng/L, depending on adsorption band chosen	[89]
Sucralose	Drinking water	HPLC/UV	Over 100 ng/L	[90]
Sucralose	Beverages	HPLC-MS/MS	10–500 ng/mL	[91]
Sucralose	Cookies	HPLC	Over 0.2 mg/kg	[92]
Acesulfame-K, aspartame, cyclamic acid, neotame, saccharin, sucralose	Beverages (soft and powdered drinks)	HPLC-ELSD	Over 1.2 μg/ml	[93]
Sucralose among its degradation products	Model and food samples	HPLC-TOP-MS	NQ	[94]
Sucralose, acesulfame-K, cyclamate, aspartame, alitame, neohesperydine dihydrochalcone, neotame	Soft drinks, energy drinks, and candy	HPLC-ELSD	Over 1.96 µg/mL (DL) 6.53 µg/mL (QL)	[95]
Sucralose, acesulfame-K	River water	LC-MS	1.836–4.766 µg/L	[96]
Acesulfame K, sucralose, saccharin, aspartame	Food, beverages, and chewing gums	HPLC, TLC	83 to 93 mg/100 mL (beverages) 82 to 155 mg/100 mL (chewing gums)	[97]
Atrazine, benzotriazole, bisphenol A, caffeine, iopromide, sucralose, TCPP. Propylparaben, carbamazepine, dexamethasone, diphenhydramine, fluoxetine, gemfibrozil, hydrochlorothiazide, hydrocortisone, prednisone, sulfamethoxazole, trimethoprim	Surface and tap water	LC-MS/MS	Over 94 ng/L	[101]
Sucralose, L-DOPA, insulin	Biological environments	HPLC	NQ	[102]
Sucralose, acesulfame K, aspartame, advantame, sodium cyclamate, neotame, saccharin, stevioside, rebaudioside	Beverages	UHPLC/MS-MS	19.4 ± 7.9 mg/mL	[103]
Sucralose and stevia	Beverages	HPLC-DAD/MS	NQ	[105]
Sucralose	Soy sauce	HPLC-DR, QuEChERS	20–1000 mg/L	[106]
Sucralose	Deproteinized food samples	SPE-HPLC-ELSD	20.26–405.2 μg/mL	[107]
Sucralose alongside its degradation products	Bacterial consortia	UYPLC-QTOF-MS	NQ	[108]

## Data Availability

No new data were created or analyzed in this study. Data sharing is not applicable to this article.
